# Antithrombotic drugs have a minimal effect on intraoperative blood loss during emergency surgery for generalized peritonitis: a nationwide retrospective cohort study in Japan

**DOI:** 10.1186/s13017-021-00374-z

**Published:** 2021-05-27

**Authors:** Tadashi Matsuoka, Nao Ichihara, Hiroharu Shinozaki, Kenji Kobayashi, Alan Kawarai Lefor, Toshimoto Kimura, Yuko Kitagawa, Yoshihiro Kakeji, Hiroaki Miyata, Junichi Sasaki

**Affiliations:** 1 Department of Emergency and Critical Care Medicine, School of Medicine, Keio University, 35 Shinanomachi, Shinkjuku, Tokyo, 164-8582 Japan; 2 Department of Surgery, Saiseikai Utsunomiya Hospital, Tochigi, Japan; 3 Department of Healthcare Quality Assessment, Graduate School of Medicine, The University of Tokyo, Tokyo, Japan; 4 Department of Surgery, Jichi Medical University, Tochigi, Japan; 5 Department of Surgery, Iwate Medical University School of Medicine, Iwate, Japan; 6 The Japanese Society of Gastroenterological Surgery, Tokyo, Japan; 7 Database Committee, The Japanese Society of Gastroenterological Surgery, Tokyo, Japan; 8 Department of Health Policy and Management, School of Medicine, Keio University, Tokyo, Japan

**Keywords:** Emergency gastrointestinal surgery, Generalized peritonitis, Antithrombotic drug, Intraoperative blood loss

## Abstract

**Background:**

The effect of antithrombotic drugs on intraoperative operative blood loss volume in patients undergoing emergency surgery for generalized peritonitis is not well defined. The purpose of this study was to investigate the effect of antithrombotic drugs on intraoperative blood loss in patients with generalized peritonitis using a nationwide surgical registry in Japan.

**Method:**

This retrospective cohort study used a nationwide surgical registry data from 2011 to 2017 in Japan. Propensity score matching for the use of antithrombotic drugs was used for the adjustment of age, gender, comorbidities, frailty, preoperative state, types of surgery, surgical approach, laboratory data, and others. The main outcome was intraoperative blood loss: comparison of intraoperative blood loss, ratio of intraoperative blood loss after adjusted for confounding factors, and variable importance of all covariates.

**Results:**

A total of 70,105 of the eligible 75,666 patients were included in this study, and 2947 patients were taking antithrombotic drugs. Propensity score matching yielded 2864 well-balanced pairs. The blood loss volume was slightly higher in the antithrombotic drug group (100 [10–349] vs 70 [10–299] ml). After adjustment for confounding factors, the use of antithrombotic drugs was related to a 1.30-fold increase in intraoperative blood loss compared to non-use of antithrombotic drugs (95% CI, 1.16–1.45). The variable importance revealed that the effect of the use of antithrombotic drugs was minimal compared with surgical approach or type of surgery.

**Conclusion:**

This study shows that while taking antithrombotic drugs is associated with a slight increase in intraoperative blood loss in patients undergoing emergency surgery for generalized peritonitis, the effect is likely of minimal clinical significance.

**Supplementary Information:**

The online version contains supplementary material available at 10.1186/s13017-021-00374-z.

## Background

The number of patients taking antithrombotic (AT) drugs is increasing along with the aging population [1]. Surgeons are more often facing situations when they have to operate on patients taking AT drugs. Emergency surgery for generalized peritonitis, which may frequently be associated with sepsis and coagulopathy, is associated with a high risk of perioperative complications, reportedly about 40~50% [2–4]. When considering surgery for patients with generalized peritonitis who also take AT drugs, there is always concern about increased intraoperative blood loss, postoperative hemorrhage, and thrombotic complications.

Clinical guidelines are inconclusive regarding the management of AT drugs for patients undergoing noncardiac surgery [5–8]. Many reports are based on studies with a small sample size or retrospective data, which may not allow conclusive statements. We previously reported that the use of AT drugs was not significantly associated with increased intraoperative blood loss in emergency gastrointestinal surgery in a single-institution study of 170 patients taking AT drugs [9]. However, including that study, there are no large-scale cohort studies to clarify the effect of AT drugs on blood loss. There are no studies to date to definitively conclude that the use of AT drugs does not affect clinically intraoperative blood loss, perioperative hemorrhagic, or thrombotic complications in patients undergoing emergency surgery for generalized peritonitis.

The purpose of this study is to investigate the effect of antithrombotic drugs on intraoperative blood loss in patients with generalized peritonitis using a nationwide surgical registry in Japan. This information is of great importance to surgeons who perform emergency gastrointestinal surgery.

## Methods

### Study design and population

This retrospective observational study used data from the National Clinical Database (NCD), which is a nationwide surgical registry in Japan that contains data on perioperative clinical characteristics and outcomes. Inclusion criteria for this study were defined as patients undergoing emergency surgery for acute generalized peritonitis from 2011 to 2017. The AT drug group was defined as patients who take AT drugs including either antiplatelet drugs or anticoagulant drugs, regardless of the use of antidotes. Data for patients with a missing value in any of the following fields were excluded: the use of AT drugs, intraoperative blood loss, date of surgery, age, or gender. Data for patients with less frequently performed procedures including highly invasive procedures such as esophagectomy or pancreatomy, surgery for non-gastrointestinal diseases, two or more major procedures in the same operation (e.g., gastrectomy and colectomy, concurrently), or surgery for trauma was excluded. This study was approved by the Institutional Review Board of Saiseikai Utsunomiya Hospital (No. 2018-15). This study is reported in accordance with the Strengthening the Reporting of Observational Studies in Epidemiology (STROBE) guidelines [10].

### Outcomes

The primary outcome of this study was intraoperative blood loss. This comprehensive investigation of the effect of the use of AT drugs on intraoperative blood loss was performed using the following three measures: comparison of intraoperative blood loss volume, ratio of intraoperative blood loss compared with non-use of AT drugs adjusted for potential confounding variables, and the variable importance of all study variables including covariates. Intraoperative blood loss was quantified by measuring suction fluid and weighing surgical gauze used for blood and fluid collection, in which fluid other than blood such as ascites was subtracted. Secondary outcomes were the incidence of severe intraoperative bleeding, defined as intraoperative blood loss of more than 1000 ml, intraoperative transfusions, bleeding complications, thrombotic complications, postoperative transfusions, in-hospital mortality, the rate of infectious complications, duration of surgery, intensive care unit (ICU) length of stay, and hospital length of stay.

### Covariates

Potential confounders included demographic factors, comorbidities, the perioperative status of the patient, procedure performed, and laboratory data (Table 1 and Table S1). Procedures performed were classified into 1 of the 12 surgical procedures listed, using the classification which would most influence the intraoperative blood loss: peritoneal lavage, gastrectomy, patch repair of peptic ulcer, other gastric surgery, colorectal resection, other colorectal surgery, small bowel obstruction surgery, other small intestinal surgery, stoma creation, appendectomy, cholecystectomy, and common bile duct surgery. Sepsis and septic shock were classified based on the sepsis-1 definition [11]. Coagulopathy was defined as both a platelet count less than 1.2 × 10^5^/μL and PT-INR more than 1.4. Acute kidney injury was defined as rapid exacerbation of creatinine to more than 3 mg/dl, as a change compared with 24-h before surgery.

**Table 1:** Demographic, clinical, and surgical characteristics

	Before Matching			After Matching		
	AT	Control	Standardized difference	AT	Control	Standardized difference
Subjects	2947	67,158		2864	2864	
Age, years (range)	77 (69–84)	70 (57–80)	− 0.561	77 (69–84)	78 (68–84)	− 0.008
Gender, Male	1884 (63.9%)	40067 (59.7%)	0.088	1815 (63.3%)	1795 (62.7%)	0.014
Body mass index (kg/m^2^)	21.5 (19.1–24.0)	21.0 (18.7–23.6)	− 0.026	21.5 (19.1–24.0)	21.3 (19.0–23.9)	− 0.014
Comorbidities						
Diabetes mellitus	767 (26.0%)	9460 (14.1%)	− 0.302	732 (25.6%)	721 (25.2%)	− 0.009
Myocardial infarction	101 (3.4%)	296 (0.4%)	− 0.218	89 (3.1%)	77 (2.7%)	− 0.025
Angina pectoris	172 (5.8%)	772 (1.1%)	− 0.257	150 (5.2%)	139 (4.9%)	− 0.018
Congestive heart failure	336 (11.4%)	1171 (1.7%)	− 0.397	299 (10.4%)	254 (8.9%)	− 0.053
Hypertension	1764 (59.9%)	21452 (31.9%)	− 0.584	1697 (59.3%)	1681 (58.7%)	− 0.011
Cerebrovascular disease	572 (19.4%)	2644 (3.9%)	− 0.497	532 (18.6%)	523 (18.3%)	− 0.008
COPD	189 (6.4%)	2045 (3.0%)	− 0.159	179 (6.3%)	166 (5.8%)	− 0.019
CKD with hemodialysis	419 (14.2%)	2172 (3.2%)	− 0.397	388 (13.5%)	370 (12.9%)	− 0.019
Advanced cancer with metastasis	150 (5.1%)	2993 (4.5%)	− 0.030	148 (5.2%)	158 (5.5%)	0.016
Long-term steroid use	312 (10.6%)	2718 (4.0%)	− 0.253	292 (10.2%)	280 (9.8%)	− 0.014
Past intervention history						
PCI	393 (13.3%)	1123 (1.7%)	− 0.454	345 (12.0%)	299 (10.4%)	− 0.051
Cardiac surgery	307 (10.4%)	853 (1.3%)	− 0.398	267 (9.3%)	224 (7.8%)	− 0.054
Peripheral vascular surgery	157 (5.3%)	295 (0.4%)	− 0.295	126 (4.4%)	96 (3.4%)	− 0.054
Activities of daily living			0.429			0.005
Independent	1901 (64.5%)	55728 (83.0%)		1873 (65.4%)	1868 (65.2%)	
Partial dependent	693 (23.5%)	7610 (11.3%)		656 (22.9%)	657 (22.9%)	
Complete dependent	353 (12.0%)	3819 (5.7%)		335 (11.7%)	339 (11.8%)	
Smoking history	397 (13.5%)	14288 (21.3%)	0.207	388 (13.5%)	394 (13.8%)	0.006
Drinking history	1083 (36.8%)	27307 (40.7%)	0.080	1046 (36.5%)	1025 (35.8%)	− 0.016
Preoperative state						
Severe Sepsis/ Septic shock	614 (20.8%)	5981 (8.9%)	0.506	579 (20.2%)	558 (19.5%)	0.024
Coagulopathy	201 (7.2%)	1209 (1.9%)	− 0.258	163 (6.0%)	163 (6.0%)	− 0.002
Acute kidney injury	324 (11.0%)	2202 (3.3%)	− 0.303	305 (10.6%)	287 (10.0%)	− 0.021
Mechanical ventilation	241 (8.2%)	1957 (2.9%)	− 0.232	226 (7.9%)	220 (7.7%)	− 0.008
Performance status			0.717			0.026
ASA-1E	53 (1.8%)	10540 (15.7%)		53 (1.9%)	60 (2.1%)	
ASA-2E	685 (23.2%)	26256 (39.1%)		681 (23.8%)	702 (24.5%)	
ASA-3E	1506 (51.1%)	22219 (33.1%)		1460 (51.0%)	1437 (50.2%)	
ASA-4E	512 (17.4%)	6035 (9.0%)		491 (17.1%)	488 (17.1%)	
ASA-5E	191 (6.5%)	2102 (3.1%)		179 (6.3%)	175 (6.1%)	
Type of surgery			0.300			0.000
Peritoneal lavage	906 (30.7%)	22468 (33.5%)		890 (31.1%)	890 (31.1%)	
Gastrectomy	17 (0.6%)	583 (0.9%)		14 (0.5%)	14 (0.5%)	
Patch repair of peptic ulcer	161 (5.5%)	7353 (10.9%)		157 (5.5%)	157 (5.5%)	
Other gastric surgery	9 (0.3%)	226 (0.3%)		8 (0.3%)	8 (0.3%)	
Colorectal resection	649 (22.0%)	11150 (16.6%)		636 (22.2%)	636 (22.2%)	
Other colorectal surgery	34 (1.2%)	708 (1.1%)		33 (1.2%)	33 (1.2%)	
Small bowel obstruction surgery	31 (1.1%)	588 (0.9%)		29 (1.0%)	29 (1.0%)	
Other small intestinal surgery	357 (12.1%)	5980 (8.9%)		344 (12.0%)	344 (12.0%)	
Stoma creation	476 (16.2%)	10002 (14.9%)		466 (16.3%)	466 (16.3%)	
Appendectomy	206 (7.0%)	6852 (10.2%)		194 (6.8%)	194 (6.8%)	
Cholecystectomy	92 (3.1%)	1089 (1.6%)		85 (3.0%)	85 (3.0%)	
Common bile duct surgery	9 (0.3%)	159 (0.2%)		8 (0.3%)	8 (0.3%)	
Surgical approach			0.193			− 0.037
Laparotomy	2688 (91.2%)	57074 (85.0%)		2611 (91.2%)	2640 (92.2%)	
Laparoscopy	259 (8.8%)	10084 (15.0%)		253 (8.8%)	224 (7.8%)	
Surgery for cancer	258 (8.8%)	6822 (10.2%)	0.048	256 (8.9%)	276 (9.6%)	0.024
Hospital volume (procedure/year)	78 (40–149)	78 (38–148)	0.029	78 (40–149)	82 (40–152)	− 0.011

Data are presented as number (percentage) or median (interquartile range)

*AT* antithrombotic drug group, *COPD* chronic obstructive pulmonary disease, *CKD* chronic kidney disease, *PCI* percutaneous coronary intervention, *ASA* American Society of Anesthesiologist

### Statistical analysis

#### Descriptive and bivariate analysis

All variables are expressed as the median (interquartile range (IQR)) or proportions. Baseline characteristics were compared between the AT drug group and the control group by standardized differences. Intraoperative blood loss was compared using the Mann-Whitney U test. The relative risk of complications and mortality were calculated among the two groups, with confidence intervals estimated assuming binomial distributions.

#### Propensity score matching

Propensity score was calculated as predicted probability of having received AT drugs preoperatively using a logistic regression model, with all covariates listed in Table 1 and Additional file 4 as independent variables, on the entire analysis dataset. Assuming balance in types of surgery and coagulopathy is of particular importance, exact matching was applied to stratify patients by these variables, followed by nearest neighbor matching within each subgroup, with a caliper of standard deviation of the propensity score multiplied by 0.25. Matching was evaluated and optimized using standardized differences as the primary measure of covariate balance.

#### Regression-adjusted effect of AT drugs on the intraoperative blood loss

Multivariable regression was applied in the matched cohort [12]. The use of AT drugs and all covariates were used as independent variables. By log-transforming intraoperative blood loss, its ratio between AT drug and control groups, adjusted for other covariates, was obtained.

#### Variable importance

Permutation variable importance, defined as a decrease in the model accuracy caused by permutation of each independent variable, was calculated to examine the relative importance of AT drugs in affecting intraoperative blood loss compared to other covariates [13, 14]. A random forest model with 1000 trees using all the independent variables was fitted for log-transformed intraoperative blood loss (ml), with zero (0) replaced with one (1).

Statistical analysis was performed by R software (version 4.0.2, 2020; R Foundation for Statistical Computing, Vienna, Austria). JMP® Pro software version 15.2.0 was also used for comparison of variables between groups (SAS Institute Inc., USA, 2020). All p-values were two-sided and p-values less than 0.05 were considered statistically significant. Details in statistical analysis were described in Additional file 1.

## Results

### Patient characteristics

**Fig. 1:**
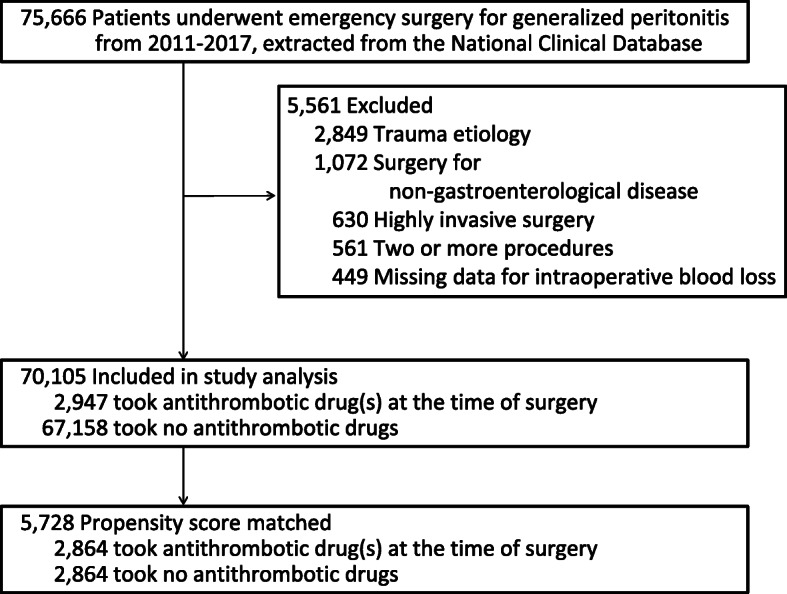
Study population

During the study period, 75,666 patients underwent emergency surgery for generalized peritonitis. After applying exclusion criteria (5561 patients), 70,105 patients remained and were analyzed as an unmatched cohort. Of these, 2947 patients (4.2%) were taking AT drugs at the time of emergency surgery. Propensity score matching selected 2864 patients who used AT drugs and 2864 patients who did not (Fig. 1, Additional file 2). Demographic, clinical and surgical characteristics before and after propensity score matching are shown in Table 1. The etiologies of generalized peritonitis are shown in Additional file 3. Before matching, patients taking AT drugs were older and had more comorbidities such as diabetes mellitus, coronary artery disease, cerebrovascular disease, and hypertension. More patients taking AT drugs also had a previous history of interventions related to cardiovascular diseases such as percutaneous coronary intervention, cardiac surgery, and peripheral vascular surgery. Preoperatively, sepsis and septic shock, and coagulopathy were seen more frequently in the AT group, and performance state was lower compared to the control group before matching. There were relatively large differences in the distribution of the types of surgical procedures performed before matching. Patients taking AT drugs were more likely to have undergone surgery of the small intestine or colon/rectum compared with patients taking no AT drugs, while patients taking no AT drugs were more likely to have undergone surgery of the stomach or appendix. Open laparotomy approach was more commonly performed for patients undergoing emergency gastrointestinal procedures on patients taking AT drugs. After matching, variables such as age, gender, comorbidities, types of surgery, and surgical approach were well balanced between the two groups. There were no laboratory data variables, which were included in the propensity score matching, with a standardized difference > 0.1 after matching (Additional file 4).

### Primary outcome

#### Intraoperative blood loss in patients taking AT drugs

**Fig. 2:**
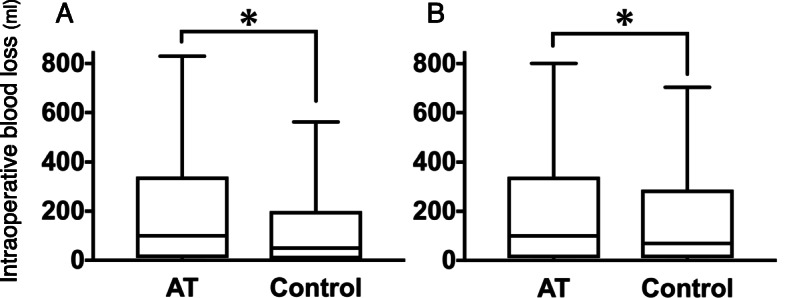
Comparison of intraoperative blood loss for antithrombotic drug use (box plot). **A** Before matching. **B** After matching. *p < 0.05 compared with the control group as analyzed using Mann-Whitney U test. AT, antithrombotic drug group. The horizontal line in the middle of each box is the median; box length is the interquartile range; whiskers represent the range of the data within the 10th and 90th percentiles

Before matching, intraoperative blood loss in the AT drug group was significantly greater than in the control group (the AT drug group vs the control group, median (IQR): 100 (10–350) vs 50 (5–200) ml). After matching, intraoperative blood loss in the AT drug group was significantly greater than in the control group (100 (10–349) vs 70 (10–299) ml) (Table 2, Fig. 2). The distribution of intraoperative blood loss by type of procedure and the differences due to taking AT drugs are shown in Additional file 5. Colon/rectal surgery and cholecystectomy had more blood loss while patch repair of peptic ulcer and appendectomy had less. For each type of procedure, the difference between the AT drug group and the control group was smaller than their interquartile ranges.

**Table 2:** The differences and the ratio of intraoperative blood loss

	AT	Control
Subjects	2864	2864
Intraoperative blood loss (ml)^b^	100 (10–349)	70 (10–299)
	Estimate (95% CI)	
Adjusted ratio of the intraoperative blood loss^a,b^	1.30 (1.16–1.45)	

Data are presented as median (interquartile range)

*AT* antithrombotic drug group, *95% CI* 95% confidential interval

^a^Compared with non-use of the antithrombotic drugs after adjustment for potential confounding variables

^b^Statistically significant

#### Adjusted ratio of intraoperative blood loss compared with not taking AT drugs

After adjustment for potential confounding variables by regression analysis in matched patients, taking AT drugs was related to a 1.30-fold increase in intraoperative blood loss compared with not taking AT drugs (95% confidence interval, 1.16–1.45) (Table 2).

#### The variable importance of AT drugs

**Fig. 3:**
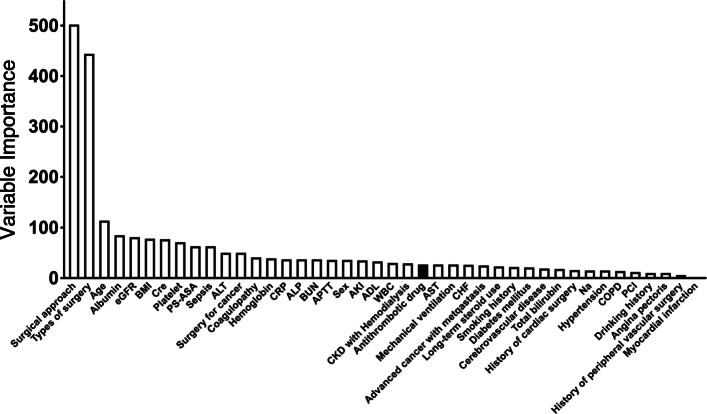
Relative importance of each covariate in predicting intraoperative blood loss by the variable importance method. Permutation variable importance was defined as a decrease in the model accuracy caused by permutation of an independent variable. All variables had statistically significant importance. eGFR, estimated glomerular filtration rate; BMI, body mass index; Cre, creatinine; PS-ASA, performance status classification by American Society of Anesthesiologist; ALT, alanine aminotransferase; CRP, C-reactive protein; ALP, alkaline phosphatase; BUN, blood urea nitrogen; APTT, activated partial thromboplastin time; AKI, acute kidney injury; ADL, activities of daily living; WBC, white blood cell; CKD, chronic kidney disease; AST, aspartate aminotransferase; CHF, congestive heart failure; Na, serum sodium; COPD, chronic obstructive pulmonary disease; PCI, percutaneous coronary intervention

**Fig. 4:**
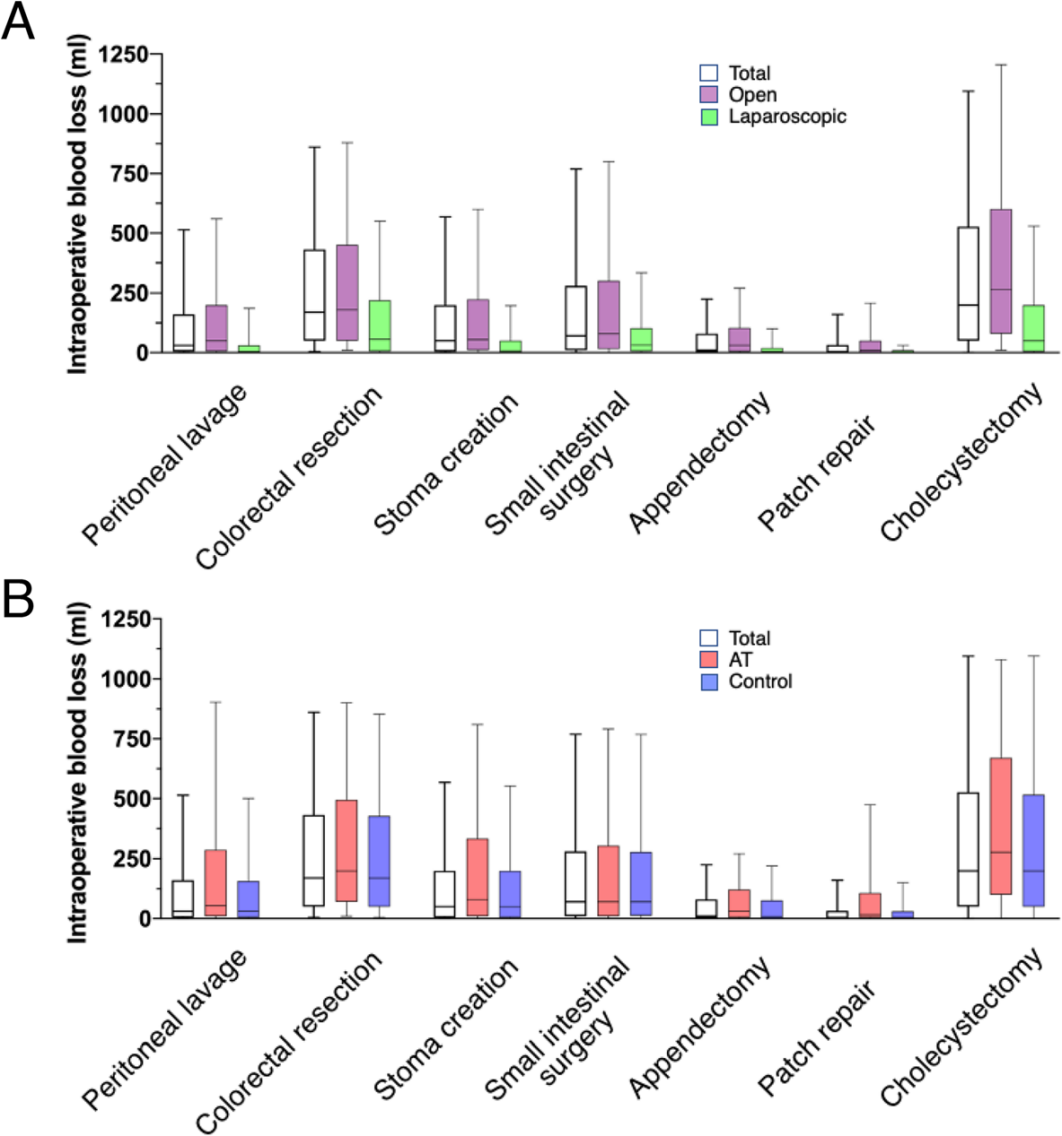
Distribution of intraoperative blood loss in the entire without matching. **A** By major procedure type and surgical approach. **B** By major procedure type and use of antithrombotic drugs. The horizontal line in the middle of each box represents the median; box length represents the interquartile range; whiskers represent the range of within the 10th and 90th percentiles

Permutation variable importance in the unmatched cohort suggested that type of surgical procedure and surgical approach have the highest impact on intraoperative blood loss among the explanatory variables. The impact of AT drugs was modest compared with type of surgery and surgical approach (Fig. 3). The distribution of intraoperative blood loss according to surgery type and surgical approach is shown in Fig. 4A. Surgery type such as colorectal surgery or cholecystectomy, and open laparotomy approach were related to more intraoperative blood loss. The distribution of intraoperative blood loss stratified by the use of AT drugs or not for every type of surgery was similar (Fig. 4B).

### Secondary outcomes

#### Bleeding and thrombosis-related surgical outcomes

Table 3 and Additional file 6 show the relationship of taking AT drugs and other outcomes examined. Before matching, variables such as the rate of bleeding and thrombotic complications, the rate of intraoperative and postoperative blood transfusions, and the rate of severe bleeding were higher among patients taking AT drugs than in the control group. After matching, variables such as the incidence of bleeding complications and the rate of intraoperative and postoperative blood transfusions were statistically significantly higher among patients taking AT drugs. There were no significant differences in other variables such as the rate of thrombotic complications or occurrence of severe bleeding between the two groups.

**Table 3:** Secondary outcomes

	AT	Control	Relative risk	95% confidential interval
Subjects	2864	2864		
Bleeding and thrombosis-related surgical outcomes				
Intraoperative severe bleeding^a^	229 (8.0%)	194 (6.8%)	1.18	0.98–1.42
Intraoperative transfusion	1020 (35.6%)	865 (30.2%)	1.18	1.09–1.27
Bleeding complication	145 (5.1%)	110 (3.8%)	1.32	1.03–1.68
Thrombotic complication	127 (4.4%)	102 (3.6%)	1.25	0.96–1.61
Postoperative transfusion	711 (24.8%)	625 (21.8%)	1.14	1.04–1.25
Other postoperative outcomes				
Mortality	664 (23.2%)	662 (23.1%)	1.00	0.91–1.10
Infectious complication				
Severe sepsis/ septic shock	567 (19.8%)	539 (18.8%)	1.05	0.95–1.17
Surgical site infection	880 (30.7%)	837 (29.2%)	1.05	0.97–1.14
Pneumonia	421 (14.7%)	407 (14.2%)	1.03	0.91–1.17
Urinary tract infection	116 (4.1%)	109 (3.8%)	1.06	0.82–1.38
Duration of operation (minutes)	129 (95–175)	125 (92–168)	-	-
ICU length of stay	5 (3–11)	5 (3–11)	-	-
Hospital length of stay	31 (16–54)	32 (18–57)	-	-

Data are presented as number (percentage) or median (interquartile range)

*AT* antithrombotic drug group, *ICU* intensive care unit

^a^Intraoperative severe bleeding was defined as intraoperative blood loss of more than 1000 ml

#### Other postoperative outcomes

Before matching, variables such as mortality, duration of operation, length of stay, and the rate of infectious complications (surgical site infection and pneumonia) were higher among patients taking AT drugs compared to the control group. After matching, only the duration of surgery was higher among patients taking AT drugs (Table 3). There were no significant differences in other variables including mortality, length of stay, or rate of infectious complications between the two groups.

## Discussion

We previously reported no significant difference in intraoperative blood loss after adjustment for confounding factors by propensity score matching in patients taking AT drugs undergoing emergency gastrointestinal surgery in a single institution [9]. In this study with a nationwide large-scale cohort, we focused on patients with generalized peritonitis, which needed surgical intervention immediately and was frequently accompanied by septic shock and coagulopathy. While this study shows a statistically significant increase in blood loss in patients taking AT drugs, the difference is minimal and the impact of intake of AT drugs on blood loss was lower compared with other factors. Other outcomes related to the use of AT drugs such as major intraoperative bleeding or the need for transfusion were also related with only a slight increase. These results suggest that AT drugs have a minimal effect on intraoperative blood loss in patients undergoing emergency surgery for generalized peritonitis after adjusting for confounding factors and are likely of minimal clinical significance. To the best of our knowledge, this is the first report to demonstrate safety for patients taking AT drugs with regard to perioperative bleeding and thrombotic complications, who undergo emergency gastrointestinal surgery for generalized peritonitis.

There is a wide range of opinions about the perioperative use of AT drugs. The American College of Surgeons’ guideline recommends cessation of aspirin for 7 to 10 days before procedures with a high risk for bleeding such as gastrointestinal surgery [5], while the Society of Thoracic Surgeons guidelines recommends continuation of aspirin monotherapy in patients undergoing noncardiac surgery [6]. The difference in the guidelines is at least partially due to the low quality of available evidence [15–19]. The incidence of difficulty obtaining intraoperative hemostasis is rarely mentioned in existing studies. The present study results shows that AT drugs were not related to difficult intraoperative hemostasis during emergency surgery for generalized peritonitis because the increase in intraoperative blood loss and rate of blood transfusions was not clinically significant although it was statistically significant. Due to study design in patients undergoing emergency surgery, a randomized controlled study cannot be carried out. The results of this nationwide study have important implications for the clinical management of patients taking AT drugs who undergo emergency gastrointestinal surgery.

Generalized peritonitis is frequently associated with systemic sepsis and is considered by some to make operative procedures more complicated, with increased blood loss due to widespread inflammation [2, 20, 21]. Increased intraoperative blood loss has unfavorable effects on immune function [22–24] and is associated with major complications or a worse prognosis [25, 26]. When performing gastrointestinal surgery on patients with generalized peritonitis, surgical outcomes including intraoperative blood loss, mortality, and postoperative morbidities tend to be worse compared to performing surgery on patients without generalized peritonitis [27, 28]. This study shows that in patients with generalized peritonitis, the effect of taking AT drugs on intraoperative blood loss and rate of blood transfusions was minimal and any increased risk of postoperative bleeding and thrombotic related complications was acceptable.

In this study, several methods of analysis gave similar results to support the conclusion that the effect of taking AT drugs was minimal. The type of surgical procedure or surgical approach, which have a high impact on intraoperative blood loss in this study, were reported as important factors associated with intraoperative blood loss in previous studies [29–32]. Therefore, less invasive procedures and surgical approach should be selected if there is no difference in mortality and morbidity expected for a particular patient. When selecting the procedure and surgical approach, taking AT drugs alone should not be a major factor in the decision-making process based on these results. For example, recent studies suggest that in patients with colorectal perforation, peritoneal lavage or laparoscopic surgery should be selected rather than open resection, if the situation permits, with no significant differences in mortality or the rate of serious complications between these procedures and surgical approaches [33–35]. Surgeons should manage patients with generalized peritonitis balancing the surgical curability of the disease and the feasibility of the selected procedure and surgical approach and do not need to place undue emphasis on the use of AT drugs in some patients.

This study has acknowledged limitations. First, this study includes patients who take all types of AT drugs including antiplatelet drugs and anticoagulant drugs because the database used in this study does not distinguish among the types of AT drugs. Different mechanisms may confer different effects on intraoperative blood loss. In addition, information about whether or not patients had therapeutic blood levels of the AT drugs at the time of emergency surgery is also unknown. Second, the use of antidotes and the timing of restarting AT drugs were at the discretion of the primary surgeon and are unknown in this study. Although the exact number of patients given vitamin K, which needs some time to normalize the PT-INR, is unknown, it would likely not be effective as an antidote for emergency surgery. Prothrombin complex concentrate and antidotes of direct oral anticoagulant were not approved yet in Japan during the study period. Therefore, we believe the effect of antidotes on the results of this study is minimal. Third, although propensity score matching is used to decrease the bias between the two groups; this study is retrospective and not blinded. Fourth, safety as an outcome is hard to quantify. We judged the primary outcome of this study as not clinically significant and “safe” because the difference and risks of outcomes are minimal. Fifth, the judgment to perform the operation and he choice of procedure is at the discretion of the individual surgeon, which could have resulted in underestimated effect of AT drugs. For a patient taking AT drugs with a high risk of bleeding, surgeons might choose a less invasive procedure, or non-operative therapy, which they would not choose if the patient did not take AT drugs. Finally, the great variety of types of surgery and surgical approaches complicates analysis of the effect of AT drugs on patient outcomes as in this study. However, when undertaking emergency surgery for patients with acute generalized peritonitis, it may be difficult to preoperatively decide the surgical procedure or approach which will finally be used. It is not unusual to change the approach according to the intraoperative findings and patients’ status. Therefore, it is important to understand the relationships between intraoperative blood loss and the use of antithrombotic drugs in patients with acute generalized peritonitis as a whole. We believe that the results of this study are of great importance to surgeons who perform emergency gastrointestinal surgery.

## Conclusion

This study revealed that while patients taking AT drugs have increased intraoperative blood loss during emergency surgery for generalized peritonitis, the effect is of minimal clinical significance in most scenarios. Therefore, surgery for patients taking AT drugs with generalized peritonitis can be performed safely although the use of AT drugs must be taken into consideration, with individualized management according to the individual condition and procedural risk stratification.

## Supplementary Information

**Additional file 1.** Title: Statistical analysis. Description: AT, antithrombotic

**Additional file 2.** Title: Mirror histogram of numbers of subjects

**Additional file 3.** Title: Etiology of generalized peritonitis

**Additional file 4.** Title: Laboratory data. Description: Data are presented as number (percentage) or median (interquartile). AT, antithrombotic drug group; AST, aspartate aminotransferase; ALT, alanine aminotransferase; ALP, alkaline phosphatase; eGFR, estimated glomerular filtration rate; CRP, C-reactive protein; APTT, activated partial thromboplastin time.

**Additional file 5.** Title: The differences of intraoperative blood loss in major types of surgery in matched cohort. Description: Data are presented as median (interquartile). AT, antithrombotic drug group.

**Additional file 6.** Title: Details of secondary outcomes. Description: Data are presented as number (percentage). AT, antithrombotic drug group.

## Data Availability

The datasets used and/or analyzed during the current study are available from the corresponding author on reasonable request.

## Abbreviations

AT: Antithrombotic
NCD: National Clinical Database
ICU: Intensive care unit
IQR: Interquartile range
