# Supplementary material for: Antithrombotic drugs have a minimal effect on intraoperative blood loss during emergency surgery for generalized peritonitis: a nationwide retrospective cohort study in Japan
Source: World J Emerg Surg. 2021 May 27;16:27. doi: 10.1186/s13017-021-00374-z (PMC8162009; doi:10.1186/s13017-021-00374-z)
Supplement: Supplementary file 1 — Additional file 1. Title: Statistical analysis. Description: AT, antithrombotic [file 13017_2021_374_MOESM1_ESM.docx]

Additional file 1. Statistical analysis

Descriptive and bivariate analysis

All variables are expressed as the median (interquartile range (IQR)) or proportions. Baseline characteristics were compared between the AT drug group and the control group by standardized differences. Intraoperative blood loss was compared using the Mann-Whitney U test. The relative risk of complications and mortality were calculated among the two groups, with confidence intervals estimated assuming binomial distributions.

Propensity score matching

Propensity score matching was used to obtain subsets of the antithrombotic drug group and the control group with negligible difference in distribution of covariates. The propensity score was calculated as the predicted probability of having received AT drugs preoperatively using a logistic regression model with no interaction terms, using all covariates listed in Table 1 and Additional file 4 as independent variables, on the entire analysis dataset. Assuming that a balance in the type of procedure and coagulopathy is of particular importance, exact matching was applied to stratify patients by these variables, followed by nearest neighbor matching within each subgroup, with a caliper of standard deviation of the propensity score multiplied by 0.25. Matching was evaluated and optimized using the standardized difference as the primary measure of covariate balance, without disclosing any intraoperative and postoperative outcomes in the two groups. For categorical variables with three or more levels, Yang and Dalton's extension of standardized difference was used[1].

Regression-adjusted effect of AT drugs on intraoperative blood loss

Multivariable regression was applied in the matched cohort to estimate the relationship of the use of AT drugs and intraoperative blood loss, adjusted for other covariates[2]. Since intraoperative blood loss had a right-skewed distribution, it was log-transformed before being used as the dependent variable. The use of AT drugs and all covariates were used as independent variables. By log-transforming intraoperative blood loss, its ratio between AT drug and control groups, adjusted for other covariates, was obtained.

Variable importance

Variable importance measures based on a random forest model were used to examine relative importance of AT drugs in determining the intraoperative blood loss compared to other covariates[3]. Random forest is an ensemble learning method, which combines numerous decision trees, with well-studied properties, and is often more accurate than a linear regression model. When compared to interpreting coefficients of a linear regression model without interaction terms, use of variable importance measures based on a random forest model has the advantage of requiring less stringent assumptions, as random forest handles non-normal distributions, non-linear correlations, and effect interactions well given sufficient number of observations. Permutation variable importance, defined as a decrease in model accuracy caused by permutation of each independent variable, was calculated to examine the relative importance of AT drugs in determining intraoperative blood loss compared to other covariates [3, 4]. A random forest model with 1,000 trees using all independent variables was fitted for log-transformed intraoperative blood loss (ml), with zero (0) replaced with one (1). "Number of variables to possibly split at in each node" and "minimal node size" were optimized through grid search to minimize the out-of-bag Gini index.

Appropriate interpretation of variable importance measures employed here requires understanding of their nature, as with any other analysis method. Particularly, it should be noted that a variable’s importance receives a lower score when the variable is correlated with any other variable examined in the analysis. Due to this, the importance of antithrombotic drugs may have been underestimated. However, this issue is common among virtually all model-based approaches for understanding the relative importance of a variable in determining an outcome, including multivariable regression.

Statistical analysis was performed using R software (version 4.0.2, 2020; R Foundation for Statistical Computing, Vienna, Austria) with the following major packages: "MatchIt" version 3.0.2 for propensity score matching, and “ranger” version 0.12.1 for calculating variable importance measures. JMP®︎ Pro software version 15.2.0 was also used for comparison of variables between groups (SAS Institute Inc., USA, 2020). All p-values were two-sided and p-values less than 0.05 were considered statistically significant.

References

1. Yang D-S, Jarrod E Dalton. A unified approach to measuring the effect size between two groups using SAS®, 2012.

2. Ho DE, Imai K, King G, Stuart EA. Matching as nonparametric preprocessing for reducing model dependence in parametric causal inference. Polit Anal. 2007;15:199-236

3. Breiman. L. Random forests. Machine Learning. 2001;45:5-32

4. Altmann A, Tolosi L, Sander O, Lengauer T. Permutation importance: a corrected feature importance measure. Bioinformatics. 2010;26:1340-7
