# Supplementary figures and images for: Antithrombotic drugs have a minimal effect on intraoperative blood loss during emergency surgery for generalized peritonitis: a nationwide retrospective cohort study in Japan
Source: World J Emerg Surg. 2021 May 27;16:27. doi: 10.1186/s13017-021-00374-z (PMC8162009; doi:10.1186/s13017-021-00374-z)

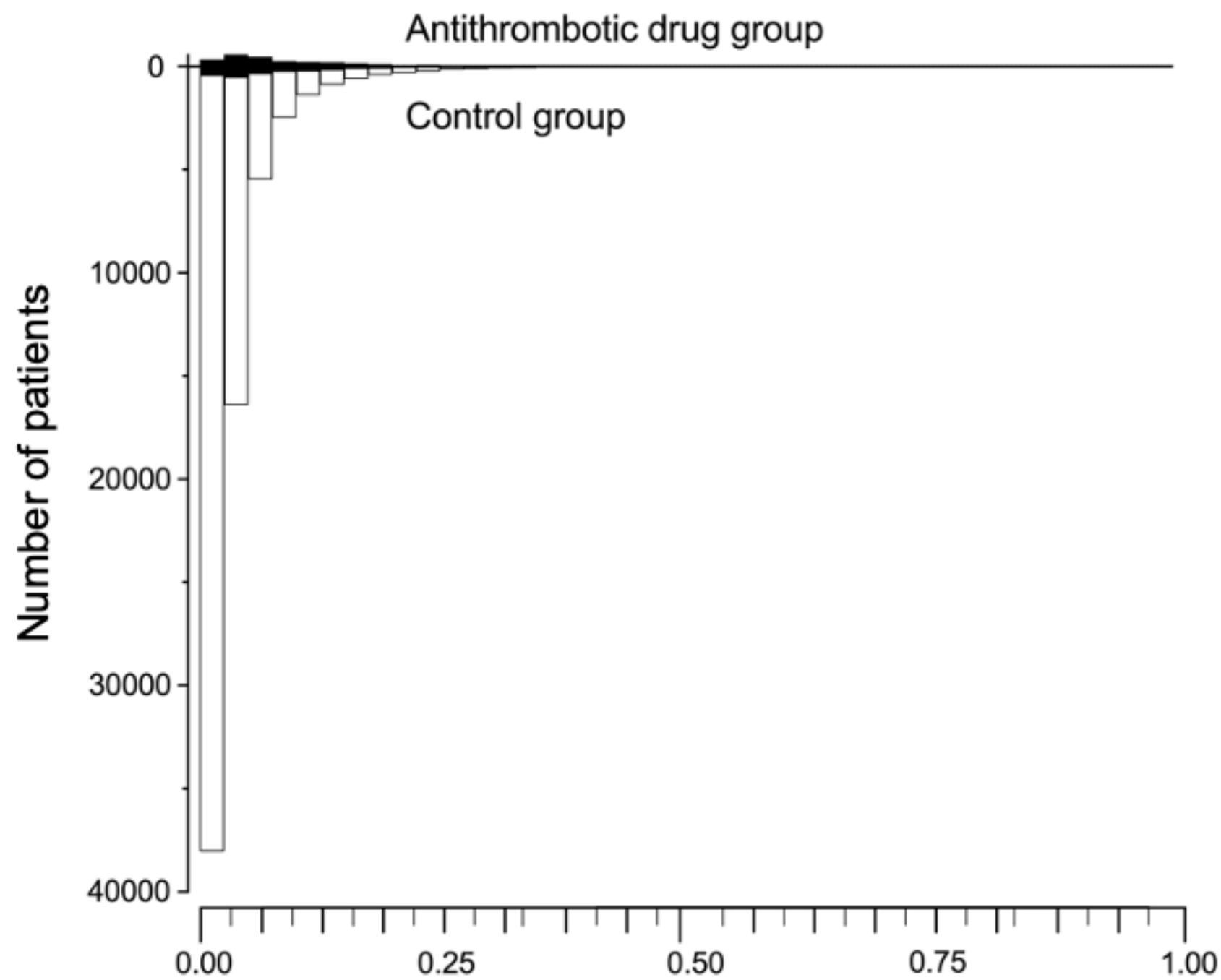

Supplement: Supplementary file 2 — Additional file 2. Title: Mirror histogram of numbers of subjects [file 13017_2021_374_MOESM2_ESM.pdf]
