# Supplementary material for: Antithrombotic drugs have a minimal effect on intraoperative blood loss during emergency surgery for generalized peritonitis: a nationwide retrospective cohort study in Japan
Source: World J Emerg Surg. 2021 May 27;16:27. doi: 10.1186/s13017-021-00374-z (PMC8162009; doi:10.1186/s13017-021-00374-z)
Supplement: Supplementary file 3 — Additional file 3. Title: Etiology of generalized peritonitis [file 13017_2021_374_MOESM3_ESM.docx]

Additional file 3. Etiology of generalized peritonitis

| Etiology of peritonitis | Before matching | |  | After matching | |
| --- | --- | --- | --- | --- | --- |
|  | Number | % |  | Number | % |
| Malignancy | 13,538 | 19.2% |  | 592 | 10.3% |
| Primary | 13,386 | 19.0% |  | 580 | 10.1% |
| Stomach | 1,383 | 2.0% |  | 102 | 1.8% |
| Duodenum | 50 | 0.1% |  | 2 | 0.0% |
| Small intestine | 293 | 0.3% |  | 26 | 0.5% |
| Colon/Rectum | 5,733 | 8.2% |  | 439 | 7.7% |
| Cecum | 87 | 0.1% |  | 26 | 0.5% |
| Ascending colon | 593 | 0.8% |  | 48 | 0.8% |
| Transverse colon | 526 | 0.8% |  | 45 | 0.8% |
| Descending colon | 312 | 0.4% |  | 30 | 0.5% |
| Sigmoid colon | 1,808 | 2.6% |  | 137 | 2.4% |
| Rectum | 2,334 | 3.3% |  | 153 | 2.7% |
| Appendix | 258 | 0.4% |  | 4 | 0.1% |
| Anus | 9 | 0.0% |  | 7 | 0.0% |
| Metastasies | 152 | 0.2% |  | 12 | 0.2% |
| Small intestine | 100 | 0.1% |  | 9 | 0.2% |
| Colon/Rectum | 52 | 0.1% |  | 3 | 0.1% |
| Peptic ulcer | 9,316 | 13.3% |  | 384 | 6.7% |
| Gastric ulcer | 3,134 | 4.5% |  | 144 | 2.5% |
| Duodenum ulcer | 5,950 | 8.5% |  | 223 | 3.9% |
| Others | 232 | 0.3% |  | 17 | 0.3% |
| Small bowel obstruction, strangulated | 4,465 | 6.4% |  | 275 | 4.8% |
| Diverticulitis | 4,573 | 6.5% |  | 523 | 9.1% |
| Lower gastrointestinal perforation | 12,616 | 18.0% |  | 1,272 | 22.2% |
| Inflammatory bowel disease | 509 | 0.7% |  | 24 | 0.4% |
| Hernia | 1,082 | 1.5% |  | 116 | 2.0% |
| Inguinal hernia | 431 | 0.6% |  | 50 | 0.9% |
| Femoral hernia | 158 | 0.2% |  | 9 | 0.2% |
| Other abdominal hernia | 493 | 0.7% |  | 57 | 1.0% |
| Cholecystitis | 1,784 | 2.5% |  | 221 | 3.9% |
| Cholangitis | 253 | 0.4% |  | 40 | 0.7% |
| Appendicitis | 10,948 | 15.6% |  | 595 | 10.4% |
| Wound dehiscence | 3,263 | 4.7% |  | 232 | 4.1% |
| Iatrogenic complication | 1,771 | 2.5% |  | 138 | 2.4% |
