# Supplementary material for: Antithrombotic drugs have a minimal effect on intraoperative blood loss during emergency surgery for generalized peritonitis: a nationwide retrospective cohort study in Japan
Source: World J Emerg Surg. 2021 May 27;16:27. doi: 10.1186/s13017-021-00374-z (PMC8162009; doi:10.1186/s13017-021-00374-z)
Supplement: Supplementary file 4 — Additional file 4. Title: Laboratory data. Description: Data are presented as number (percentage) or median (interquartile). AT, antithrombotic drug group; AST, aspartate aminotransferase; ALT, alanine aminotransferase; ALP, alkaline phosphatase; eGFR, estimated glomerular filtration rate; CRP, C-reactive protein; APTT, activated partial thromboplastin time. [file 13017_2021_374_MOESM4_ESM.docx]

Additional file 4. Laboratory data

|  | Before Matching | | |  | After Matching | | |
| --- | --- | --- | --- | --- | --- | --- | --- |
|  | AT | Control | Standardized difference |  | AT | Control | Standardized difference |
| White blood cell count (/μL) |  |  | 0.072 |  |  |  | 0.022 |
| <3500 | 439 (15.1%) | 8645 (13.0%) |  |  | 428 (15.1%) | 409 (14.4%) |  |
| 3500≦, ≦9000 | 1010 (34.7%) | 22290 (33.5%) |  |  | 983 (34.7%) | 977 (34.4%) |  |
| 9000< | 1464 (50.3%) | 35506 (53.4%) |  |  | 1419 (50.1%) | 1450 (51.1%) |  |
| Hemoglobin (g/dL) |  |  | 0.304 |  |  |  | 0.022 |
| male, <13.5; female, <11.5 | 1849 (65.9%) | 32497 (50.5%) |  |  | 1778 (65.3%) | 1770 (64.5%) |  |
| male, 13.5≦; female, 11.5≦ | 957 (34.1%) | 31869 (49.5%) |  |  | 946 (34.7%) | 976 (35.5%) |  |
| Platelet count (x10^5/μL) |  |  | 0.296 |  |  |  | 0.005 |
| <1.5 | 749 (26.7%) | 9357 (14.5%) |  |  | 700 (25.7%) | 699 (25.5%) |  |
| 1.5≦, ≦3.5 | 1796 (64.1%) | 45951 (71.4%) |  |  | 1764 (64.9%) | 1770 (64.6%) |  |
| 3.5< | 256 (9.1%) | 9017 (14.0%) |  |  | 255 (9.4%) | 269 (9.8%) |  |
| Albumin (g/dL) |  |  | 0.301 |  |  |  | 0.046 |
| <4.0 | 2304 (87.4%) | 45227 (75.0%) |  |  | 2229 (87.1%) | 2260 (87.1%) |  |
| 4.0≦ | 333 (12.6%) | 15073 (25.0%) |  |  | 329 (12.9%) | 336 (12.9%) |  |
| Total bilirubin (mg/dL) |  |  | 0.087 |  |  |  | 0.049 |
| ≦1.2 | 1904 (79.8%) | 46027 (82.5%) |  |  | 1850 (79.9%) | 1915 (81.0%) |  |
| 1.2< | 483 (20.2%) | 9725 (17.4%) |  |  | 465 (20.1%) | 451 (19.1%) |  |
| AST (U/L) |  |  | 0.138 |  |  |  | 0.052 |
| ≦35 | 1915 (79.3%) | 47933 (84.7%) |  |  | 1871 (79.8%) | 1940 (81.3%) |  |
| 35< | 502 (20.8%) | 8628 (15.3%) |  |  | 475 (20.2%) | 445 (18.7%) |  |
| ALT (U/L) |  |  | 0.030 |  |  |  | 0.023 |
| ≦35 | 2343 (83.9%) | 54208 (84.7%) |  |  | 2283 (84.2%) | 2309 (84.6%) |  |
| 35< | 449 (16.1%) | 9786 (15.3%) |  |  | 427 (15.8%) | 419 (15.4%) |  |
| ALP (U/L) |  |  | 0.085 |  |  |  | 0.063 |
| ≦340 | 1872 (83.6%) | 45355 (86.2%) |  |  | 1817 (82.2%) | 1901 (85.7%) |  |
| 340< | 368 (16.4%) | 7284 (13.8%) |  |  | 355 (16.3%) | 317 (14.3%) |  |
| Blood urea nitrogen (mg/dL) |  |  | 0.460 |  |  |  | 0.019 |
| ≦20 | 963 (34.3%) | 36642 (57.0%) |  |  | 951 (34.8%) | 973 (35.6%) |  |
| 20< | 1849 (65.8%) | 27578 (42.9%) |  |  | 1778 (65.2%) | 1765 (64.5%) |  |
| Serum creatinine | 1.17 (0.79-2.08) | 0.84 (0.65-1.21) | -0.326 |  | 1.15 (0.79-2.04) | 1.12 (0.77-2.02) | -0.029 |
| eGFR (mL/min per 1.73m^2^) | 54.5 (28.1-85.8) | 84.1 (52.4-112.5) | 0.431 |  | 55.6 (28.4-86.1) | 58.1 (28.5-89.8) | 0.022 |
| Serum sodium |  |  | 0.083 |  |  |  | 0.051 |
| <138 | 740 (32.3%) | 16648 (31.0%) |  |  | 723 (32.5%) | 685 (30.4%) |  |
| 138≦, ≦146 | 1496 (65.4%) | 36454 (67.8%) |  |  | 1450 (65.2%) | 1523 (67.5%) |  |
| 146< | 53 (2.3%) | 661 (1.2%) |  |  | 51 (2.3%) | 49 (2.2%) |  |
| CRP (mg/dL) |  |  | 0.156 |  |  |  | 0.003 |
| ≦0.1 | 195 (7.0%) | 7416 (11.6%) |  |  | 192 (7.1%) | 194 (7.1%) |  |
| 0.1< | 2607 (93.0%) | 56434 (88.4%) |  |  | 2530 (92.9%) | 2547 (92.9%) |  |
| APTT (sec) |  |  | 0.306 |  |  |  | 0.089 |
| <30 | 293 (14.3%) | 10097 (20.8%) |  |  | 291 (14.6%) | 277 (13.4%) |  |
| 30≦, ≦40 | 1134 (55.2%) | 32312 (66.6%) |  |  | 1120 (56.1%) | 1245 (60.2%) |  |
| 40< | 628 (30.6%) | 6142 (12.7%) |  |  | 586 (29.3%) | 546 (26.4%) |  |

Data are presented as number (percentage) or median (interquartile).

AT, antithrombotic drug group; AST, aspartate aminotransferase; ALT, alanine aminotransferase; ALP, alkaline phosphatase; eGFR, estimated glomerular filtration rate; CRP, C-reactive protein; APTT, activated partial thromboplastin time.
