# Supplementary material for: Antithrombotic drugs have a minimal effect on intraoperative blood loss during emergency surgery for generalized peritonitis: a nationwide retrospective cohort study in Japan
Source: World J Emerg Surg. 2021 May 27;16:27. doi: 10.1186/s13017-021-00374-z (PMC8162009; doi:10.1186/s13017-021-00374-z)
Supplement: Supplementary file 5 — Additional file 5. Title: The differences of intraoperative blood loss in major types of surgery in matched cohort. Description: Data are presented as median (interquartile). AT, antithrombotic drug group. [file 13017_2021_374_MOESM5_ESM.docx]

Additional file 5. The differences of intraoperative blood loss in major types of surgery in matched cohort

|  | AT | | Control | |
| --- | --- | --- | --- | --- |
| Type of surgery | Number | Blood loss | Number | Blood loss |
| All procedure | 2864 | 100 (349-10) | 2864 | 70 (299-10) |
| Peritoneal lavage | 890 | 53 (273-10) | 890 | 50 (200-5) |
| Patch repair of peptic ulcer | 157 | 20 (108-5) | 157 | 10 (70-0) |
| Colorectal resection | 636 | 200 (490-69) | 636 | 175 (432-50) |
| Small intestinal resection | 344 | 75 (304-10) | 344 | 75 (300-10) |
| Stoma creation | 466 | 80 (334-10) | 466 | 57 (236-5) |
| Cholecystectomy | 85 | 271 (658-100) | 85 | 220 (676-70) |
| Appendectomy | 194 | 30 (130-5) | 194 | 25 (103-5) |

Data are presented as median (interquartile).

AT, antithrombotic drug group.
