# Supplementary material for: Antithrombotic drugs have a minimal effect on intraoperative blood loss during emergency surgery for generalized peritonitis: a nationwide retrospective cohort study in Japan
Source: World J Emerg Surg. 2021 May 27;16:27. doi: 10.1186/s13017-021-00374-z (PMC8162009; doi:10.1186/s13017-021-00374-z)
Supplement: Supplementary file 6 — Additional file 6. Title: Details of secondary outcomes. Description: Data are presented as number (percentage). AT, antithrombotic drug group. [file 13017_2021_374_MOESM6_ESM.docx]

Additional file 6. Details of secondary outcomes

|  | AT | Control |
| --- | --- | --- |
| Subjects | 2864 | 2864 |
| Intraoperative transfusion | 1020 (35.6%) | 865 (30.2%) |
| Red blood cell | 811 (28.3%) | 740 (25.8%) |
| Fresh frozen plasma | 642 (22.4%) | 421 (14.7%) |
| Platelet | 99 (3.5%) | 88 (3.1%) |
| Bleeding complication, all | 145 (5.1%) | 110 (3.8%) |
| Reoperation due to bleeding | 47 (1.6%) | 28 (1.0%) |
| Intraabdominal bleeding | 59 (2.1%) | 29 (1.0%) |
| Gastrointestinal bleeding | 67 (2.3%) | 56 (2.0%) |
| Cerebral bleeding | 11 (0.4%) | 10 (0.3%) |
| Thrombotic complication, all | 127 (4.4%) | 102 (3.6%) |
| Myocardial infarction | 20 (0.7%) | 11 (0.4%) |
| Pulmonary embolism | 17 (0.6%) | 11 (0.4%) |
| Cerebral infarction | 60 (2.1%) | 50 (1.7%) |
| Deep vein thrombosis | 47 (1.6%) | 38 (1.3%) |

Data are presented as number (percentage).

AT, antithrombotic drug group.
